# Supplementary material for: The Systems Biology Research Tool: evolvable open-source software
Source: BMC Syst Biol. 2008 Jun 29;2:55. doi: 10.1186/1752-0509-2-55 (PMC2446383; doi:10.1186/1752-0509-2-55)
Supplement: Additional file 1 — SBRT Archive. An archive of the current version of the Systems Biology Research Tool. [file 1752-0509-2-55-S1.zip › sbrt-1.4.0/doc/developers_guide/api/sbrt/shell/mng/fba/InitialPointManager.html]

InitialPointManager


|  |  |  |
| --- | --- | --- |
| **Overview** \| **Package** \| **Class** \| **Use** \| **Tree** \| **Deprecated** \| **Index** \| **Help** | |  |
| **PREV CLASS**   **NEXT CLASS** | **FRAMES**    **NO FRAMES**     **All Classes** |  |
| SUMMARY: NESTED \| FIELD \| CONSTR \| METHOD | DETAIL: FIELD \| CONSTR \| METHOD |  |


---


## sbrt.shell.mng.fba Class InitialPointManager

```
java.lang.Object
  sbrt.shell.mng.AbstractProcessManager
      sbrt.shell.mng.fba.FbaManager
          sbrt.shell.mng.fba.InitialPointManager
```

**All Implemented Interfaces:**: KernelProcessManager<java.lang.String,java.lang.String>, ProcessManager<java.lang.String,java.lang.String>

---

``` public final class InitialPointManager extends FbaManager ```

This class is used to manage initial point experiments.

**Author:**
:   This class was written and documented by Jeremiah Wright while in the
    Wagner lab.

---

| **Field Summary** | |
| --- | --- |
| `static double` | `DEFAULT_P`             The default P intercept value. |
| `static java.lang.String` | `FLUX_INTERVAL_FILE`             The keyword used to denote the name of the file containing the set of flux intervals. |
| `static UnmodifiableSet<java.lang.String>` | `optionalKeywords`             The set of keywords understood, but not required, by instances of this class. |
| `static java.lang.String` | `P_MAX`             The keyword used to denote the P intercept (the value of positioning variables at the edge of the convex poytope). |
| `static java.lang.String` | `P_VECTOR_FILE_NAME`             The keyword used to denote the name of the C vector output file. |
| `static UnmodifiableSet<java.lang.String>` | `requiredKeywords`             The set of keywords required to be present in the `ManagerInput` objects supplied to instances of this class. |

**Fields inherited from class sbrt.shell.mng.fba.FbaManager**

`CONSTRAINT_TOLERANCE, DEFAULT_CONSTRAINT_TOLERANCE, FLUX_VECTOR_FILE, FLUX_VECTOR_FILE_NAME, RXN_CATALYST_FILE, RXN_FILE, SYSTEM_SOLUTION_FILE`

**Fields inherited from class sbrt.shell.mng.AbstractProcessManager**

`ALGORITHM, COMPLETED, DATA_HEADERS, DEV_NULL, ELAPSED_TIME, FILE_FORMAT, INPUT_FILE, INPUT_FILE_FORMAT, INPUT_FILE_NAME_FILE, ITERATIONS, OUTPUT_FILE_FORMAT, OUTPUT_FILE_NAME, OUTPUT_FILE_NAME_FILE, PERCENT, PERCENT_COMPLETED, SEED, ZERO_CUTOFF`


**Constructor Summary**

`InitialPointManager()`


| **Method Summary** | |
| --- | --- |
| `void` | `execute(java.io.PrintWriter out)`             Executes the process this process manager controls, and writes the status or results of this process to the provided print writer. |
| `static FbaOptComputer` | `getComputer(ManagerInput<java.lang.String,java.lang.String> input, java.io.PrintWriter out)`             Creates and returns an FBA optimization computer based on the provided input. |
| `static MapOutputFile<java.lang.String,java.lang.Double>` | `getCVectorFile(ManagerInput<java.lang.String,java.lang.String> input, java.io.PrintWriter out, Fluxome fluxome)`             Returns an empty output file that is suitable for storing a single C vector with the name denoted by `P_VECTOR_FILE_NAME` in the provided input. |
| `static IrrevRxnFluxome` | `getFluxome(ManagerInput<java.lang.String,java.lang.String> input, java.io.PrintWriter out, double tolerance)`             Creates and returns a fluxome based on the provided input. |
| `static MapOutputFile<java.lang.String,java.lang.Double>` | `getFluxVectorFile(ManagerInput<java.lang.String,java.lang.String> input, java.io.PrintWriter out, Fluxome fluxome)`             Returns an empty output file that is suitable for storing a single flux vector. |
| `static FluxConstraints` | `getIntervals(ManagerInput<java.lang.String,java.lang.String> input, java.io.PrintWriter out, Fluxome fluxome)`             Opens the file denoted by `FLUX_INTERVAL_FILE` in the provided manager input and returns the set of flux intervals it contains. |
| `static FluxConstraints` | `getIntervals(java.lang.String fileName, Fluxome fluxome)`             Returns the set of flux intervals contained in the file with the provided name. |
| `UnmodifiableSet<java.lang.String>` | `getOptionalKeys()`             Returns the set of keywords the provided `ManagerInput` object does not necessarily have to contain. |
| `static double` | `getPIntercept(ManagerInput<java.lang.String,java.lang.String> input, java.io.PrintWriter out)`             Returns the parsed value for `P_MAX` in the provided input. |
| `InitialPointGenerator` | `getProcess()`             Returns the process this process manager controls. |
| `UnmodifiableSet<java.lang.String>` | `getRequiredKeys()`             Returns the set of keywords the provided `ManagerInput` object must contain. |
| `void` | `setInput(ManagerInput<java.lang.String,java.lang.String> input, java.io.PrintWriter out)`             Uses the provided input to prepare this process manager for execution. |

**Methods inherited from class sbrt.shell.mng.fba.FbaManager**

`getCatalysts, getCatalysts, getConstraintTolerance, getConstraintTolerance, getFluxome, getFluxome, getFluxome, getFluxome, getFluxome, getFluxomeSolution, getMultiFluxVectorOutputFile, getMultiFluxVectorOutputFile, getRxnNameExprFormat, getRxnNameHeaders, getRxnNameHeaders, getSingleFluxVectorOutputFile, getSingleFluxVectorOutputFile, printFluxomeInfo, writeFluxome, writeFluxome`

**Methods inherited from class sbrt.shell.mng.AbstractProcessManager**

`check, closeFile, closeRm, execute, getFileFormat, getFileFormat, getFileNameSource, getIterations, getSeed, getTextFileFormat, getTextFileFormat, getZeroCutoff, getZeroCutoff, parseInt, parseLong, printProgress, printProgress, printProgress, runProcess, setInput`

**Methods inherited from class java.lang.Object**

`clone, equals, finalize, getClass, hashCode, notify, notifyAll, toString, wait, wait, wait`

**Methods inherited from interface sbrt.shell.mng.ProcessManager**

`check, execute, setInput`

**Field Detail**

### P\_MAX

```
public static final java.lang.String P_MAX
```

:   The keyword used to denote the P intercept (the value of positioning
    variables at the edge of the convex poytope).

    **See Also:**: Constant Field Values

---


### DEFAULT\_P

```
public static final double DEFAULT_P
```

:   The default P intercept value.

    **See Also:**: Constant Field Values

---


### P\_VECTOR\_FILE\_NAME

```
public static final java.lang.String P_VECTOR_FILE_NAME
```

:   The keyword used to denote the name of the C vector output file.

    **See Also:**: Constant Field Values

---


### FLUX\_INTERVAL\_FILE

```
public static final java.lang.String FLUX_INTERVAL_FILE
```

:   The keyword used to denote the name of the file containing the set of
    flux intervals.

    **See Also:**: Constant Field Values

---


### requiredKeywords

```
public static final UnmodifiableSet<java.lang.String> requiredKeywords
```

:   The set of keywords required to be present in the `ManagerInput`
    objects supplied to instances of this class.

---


### optionalKeywords

```
public static final UnmodifiableSet<java.lang.String> optionalKeywords
```

:   The set of keywords understood, but not required, by instances of this
    class.


**Constructor Detail**

### InitialPointManager

```
public InitialPointManager()
```


**Method Detail**

### getPIntercept

```
public static double getPIntercept(ManagerInput<java.lang.String,java.lang.String> input,
                                   java.io.PrintWriter out)
```

:   Returns the parsed value for `P_MAX` in the provided input.

    :   **Parameters:**: `input` - the input whose `P_INTERCEPT` value is to be parsed.: `out` - the print writer to which a status message will be printed. **Returns:**: the parsed numerical P intercept value; or `DEFAULT_P` if no value for `P_INTERCEPT` exists. **Throws:**: `ApplicationException` - if the parsed intercept is infinite, NaN, or < 10.

---


### getFluxVectorFile

```
public static MapOutputFile<java.lang.String,java.lang.Double> getFluxVectorFile(ManagerInput<java.lang.String,java.lang.String> input,
                                                                                 java.io.PrintWriter out,
                                                                                 Fluxome fluxome)
```

:   Returns an empty output file that is suitable for storing a single flux
    vector. The values for `FbaManager.FLUX_VECTOR_FILE_NAME` and
    `AbstractProcessManager.DATA_HEADERS` in the provided input are
    parsed and used to call the method
    `FbaManager.getSingleFluxVectorOutputFile(String, FileFormat, Set, Fluxome)`.

    :   **Parameters:**: `input` - the input containing values for the listed keywords.: `out` - the print writer to which status messages will be printed.: `fluxome` - the fluxome for which the data headers correspond. **Returns:**: an empty output file that is suitable for storing a single flux vector. **See Also:**: `FbaManager.getRxnNameHeaders(ManagerInput, Fluxome)`

---


### getCVectorFile

```
public static MapOutputFile<java.lang.String,java.lang.Double> getCVectorFile(ManagerInput<java.lang.String,java.lang.String> input,
                                                                              java.io.PrintWriter out,
                                                                              Fluxome fluxome)
```

:   Returns an empty output file that is suitable for storing a single C
    vector with the name denoted by `P_VECTOR_FILE_NAME` in the
    provided input.

    :   **Parameters:**: `input` - the input containing values for the listed keywords.: `out` - the print writer to which status messages will be printed.: `fluxome` - the fluxome for which the data headers correspond. **Returns:**: an empty output file that is suitable for storing a single C vector.

---


### getComputer

```
public static FbaOptComputer getComputer(ManagerInput<java.lang.String,java.lang.String> input,
                                         java.io.PrintWriter out)
```

:   Creates and returns an FBA optimization computer based on the provided
    input. The values for `FbaManager.RXN_FILE`, `FLUX_INTERVAL_FILE`,
    `FbaManager.CONSTRAINT_TOLERANCE`,
    `FbaOptManager.SAFETY_LEVEL`, `ProgramSolverFactory.SOLVER`,
    and `ProgramSolverFactory.PARAM_FILE` are parsed and combined to
    create the returned optimization computer.

    :   **Parameters:**: `input` - the input containing values for the listed keywords.: `out` - the print writer to which status messages will be printed. **Returns:**: an FBA optimization computer based on the provided input. **See Also:**: `FbaManager.getFluxome(ManagerInput, PrintWriter)`, `FbaOptManager.getSafetyLevel(ManagerInput, PrintWriter)`, `FbaManager.getConstraintTolerance(ManagerInput, PrintWriter)`, `ProgramSolverFactory.make(ManagerInput, PrintWriter)`

---


### getFluxome

```
public static IrrevRxnFluxome getFluxome(ManagerInput<java.lang.String,java.lang.String> input,
                                         java.io.PrintWriter out,
                                         double tolerance)
```

:   Creates and returns a fluxome based on the provided input. The values for
    `FbaManager.RXN_FILE` and `FLUX_INTERVAL_FILE` are parsed and combined
    to create the returned fluxome.

    :   **Parameters:**: `input` - the input containing values for the listed keywords.: `out` - the print writer to which status messages will be printed.: `tolerance` - the constraint tolerance. **Returns:**: a fluxome based on the provided input. **See Also:**: `FbaManager.getFluxome(ManagerInput, PrintWriter)`, `getIntervals(ManagerInput, PrintWriter, Fluxome)`, `Fluxome.replaceConstraints(FluxConstraints, double)`

---


### getIntervals

```
public static FluxConstraints getIntervals(ManagerInput<java.lang.String,java.lang.String> input,
                                           java.io.PrintWriter out,
                                           Fluxome fluxome)
```

:   Opens the file denoted by `FLUX_INTERVAL_FILE` in the provided
    manager input and returns the set of flux intervals it contains.

    :   **Parameters:**: `input` - the input containing a value for `FLUX_INTERVAL_FILE`.: `out` - the print writer to which a status message will be printed.: `fluxome` - the fluxome to which the flux intervals correspond. **Returns:**: the set of flux intervals contained in the file indicated by `#FLUX_INTERVAL_FILE` in the provided input. **Throws:**: `ApplicationException` - if the file does not contain a flux interval for each reaction in the provided fluxome, or if an interval in unbounded. **See Also:**: `getIntervals(String, Fluxome)`

---


### getIntervals

```
public static FluxConstraints getIntervals(java.lang.String fileName,
                                           Fluxome fluxome)
```

:   Returns the set of flux intervals contained in the file with the provided
    name. This file must have the format of a row-oriented map file. The
    reaction names in this file must be parsable using
    `RxnNameVerifier`, and the intervals must be parsable using
    `BoundedIntervalFormatV1`. All reaction names from the provided
    fluxome must be present.

    :   **Parameters:**: `fileName` - the name of the file to be read.: `fluxome` - the fluxome to which the flux intervals correspond. **Returns:**: the set of flux intervals contained in the file with the provided name. **Throws:**: `ApplicationException` - if the file does not contain a flux interval for each reaction in the provided fluxome, or if an interval in unbounded.: `FormatException` - if any part of the file cannot be parsed.: `java.lang.NullPointerException` - if any argument is `null`. **See Also:**: `RomiFile`

---


### getRequiredKeys

```
public UnmodifiableSet<java.lang.String> getRequiredKeys()
```

:   Returns the set of keywords the provided `ManagerInput` object must
    contain.

    :   **Returns:**: the set of keywords the provided `ManagerInput` object must contain.

---


### getOptionalKeys

```
public UnmodifiableSet<java.lang.String> getOptionalKeys()
```

:   Returns the set of keywords the provided `ManagerInput` object does
    not necessarily have to contain.

    :   **Returns:**: the set of keywords the provided `ManagerInput` object does not necessarily have to contain.

---


### getProcess

```
public InitialPointGenerator getProcess()
```

:   Returns the process this process manager controls.

    :   **Returns:**: the process this process manager controls. **Throws:**: `java.lang.IllegalStateException` - if no input was provided to this process manager via `AbstractProcessManager.setInput(ManagerInput)` or `setInput(ManagerInput, PrintWriter)`

---


### setInput

```
public void setInput(ManagerInput<java.lang.String,java.lang.String> input,
                     java.io.PrintWriter out)
```

:   Uses the provided input to prepare this process manager for execution. As
    the input is processed, informative messages are printed to the provided
    print writer.

    :   **Parameters:**: `input` - all of the information required to prepare this process manager for execution.: `out` - the print writer to which all messages will be printed.

---


### execute

```
public void execute(java.io.PrintWriter out)
```

:   Executes the process this process manager controls, and writes the status
    or results of this process to the provided print writer.

    :   **Parameters:**: `out` - the print writer where status messages will be printed. **Throws:**: `java.lang.IllegalStateException` - if no input was provided to this process manager via `AbstractProcessManager.setInput(ManagerInput)` or `setInput(ManagerInput, PrintWriter)`


---


|  |  |  |
| --- | --- | --- |
| **Overview** \| **Package** \| **Class** \| **Use** \| **Tree** \| **Deprecated** \| **Index** \| **Help** | |  |
| **PREV CLASS**   **NEXT CLASS** | **FRAMES**    **NO FRAMES**     **All Classes** |  |
| SUMMARY: NESTED \| FIELD \| CONSTR \| METHOD | DETAIL: FIELD \| CONSTR \| METHOD |  |


---
